# Natural *Wolbachia* infections are common in the major malaria vectors in Central Africa

**DOI:** 10.1111/eva.12804

**Published:** 2019-06-11

**Authors:** Diego Ayala, Ousman Akone‐Ella, Nil Rahola, Pierre Kengne, Marc F. Ngangue, Fabrice Mezeme, Boris K. Makanga, Martha Nigg, Carlo Costantini, Frédéric Simard, Franck Prugnolle, Benjamin Roche, Olivier Duron, Christophe Paupy

**Affiliations:** ^1^ MIVEGEC, IRD, CNRS Université de Montpellier Montpellier France; ^2^ CIRMF Franceville Gabon; ^3^ ANPN Libreville Gabon; ^4^ UMMISCO, IRD Montpellier France

**Keywords:** *Anopheles*, co‐evolution, disease control, diversity, *Wolbachia*

## Abstract

During the last decade, the endosymbiont bacterium *Wolbachia* has emerged as a biological tool for vector disease control. However, for long time, it was believed that *Wolbachia* was absent in natural populations of *Anopheles*. The recent discovery that species within the *Anopheles gambiae* complex host *Wolbachia* in natural conditions has opened new opportunities for malaria control research in Africa. Here, we investigated the prevalence and diversity of *Wolbachia* infection in 25 African *Anopheles* species in Gabon (Central Africa). Our results revealed the presence of *Wolbachia* in 16 of these species, including the major malaria vectors in this area. The infection prevalence varied greatly among species, confirming that sample size is a key factor to detect the infection. Moreover, our sequencing and phylogenetic analyses showed the important diversity of *Wolbachia* strains that infect *Anopheles*. Co‐evolutionary analysis unveiled patterns of *Wolbachia* transmission within some *Anopheles* species, suggesting that past independent acquisition events were followed by co‐cladogenesis. The large diversity of *Wolbachia* strains that infect natural populations of *Anopheles* offers a promising opportunity to select suitable phenotypes for suppressing *Plasmodium* transmission and/or manipulating *Anopheles* reproduction, which in turn could be used to reduce the malaria burden in Africa.

## 1 INTRODUCTION

Malaria still affects millions of people and is the cause of thousands of deaths worldwide, although sub‐Saharan Africa pays the highest tribute (WHO, 2018). Currently, vector control measures (e.g., insecticide‐treated bed nets or indoor residual sprays) are the largest contributors to malaria eradication (Bhatt et al., 2015). If these interventions are maintained or increased, malaria burden should be drastically reduced in Africa before 2030 (Griffi et al., 2016). These predictions are based on the constant effectiveness of these methods. However, the spread of insecticide resistance (Ranson & Lissenden, 2016) and vector behavioural changes related to the massive use of bed nets (Pates & Curtis, 2005) might challenge malaria eradication in the coming decades. Therefore, it is vital to develop alternative and non‐insecticide‐based control strategies for malaria control, at it has been promoted by the Global Technical Strategy form Malaria 2016–2030, which look for “reducing global malaria incidence and mortality rates by at least 90% by 2030” (Newby et al., 2016; WHO, 2015).

Several methods have been proposed to accompany or replace the use of synthetic insecticides (McGraw & O'Neill, 2013). Among them, the use of the maternally inherited *Wolbachia* bacteria (α‐proteobacteria, Anaplasmataceae family) has emerged as a promising alternative biological tool for fighting malaria and other vector‐borne diseases (Bourtzis et al., 2014; Hoffmann, Ross, & Rasic, 2015; Iturbe‐Ormaetxe, Walker, & Neill, 2011; Kambris, Cook, Phuc, & Sinkins, 2009; McGraw & O'Neill, 2013). This bacterium exhibits a large spectrum of interactions with its hosts: from mutualism and commensalism to parasitism (Werren, Baldo, & Clark, 2008). Moreover, *Wolbachia* can invade mosquito populations and/or prevent vector‐borne infections in some of the most important mosquito vectors (Dodson et al., 2014; Hoffmann et al., 2015; Iturbe‐Ormaetxe et al., 2011). Indeed, *Aedes aegypti* populations that were artificially infected with *Wolbachia* have been successfully used to suppress dengue transmission in laboratory conditions and have been released in natural populations of this mosquito (Hoffmann et al., 2011; Schmidt et al., 2017). Similarly, laboratory studies showed that infection of *Anopheles* (the vector of human malaria) with *Wolbachia* strains has a negative impact on the transmission of *Plasmodium* parasites (Bian et al., 2013; Hughes, Koga, Xue, Fukatsu, & Rasgon, 2011; Kambris et al., 2010), providing a relevant alternative for malaria control. Unfortunately, only one stable transfected *Wolbachia* colony has been described in *Anopheles stephensi* (Bian et al., 2013). Therefore, data on the use *Wolbachia* for *Anopheles* control remain scarce and mainly concern experimental studies in laboratory conditions (Bian et al., 2013; Hughes, Vega‐Rodriguez, Xue, & Rasgon, 2012), due to technical (i.e., egg microinjection) and biological (i.e., competitive exclusion with the bacterium *Asaia*) difficulties in carrying out transinfections in *Anopheles*, despite multiple assays (Hughes, Dodson, et al., 2014; Jeffries, Golovko, et al., 2018; Jeffries, Lawrence, et al., 2018; Rossi et al., 2015). For a long time, it was assumed that *Wolbachia* was absent in natural populations of *Anopheles* (Hughes, Dodson, et al., 2014). However, in the last few years, three studies reported that *Anopheles gambiae, Anopheles coluzzii* and *Anopheles arabiensis* (three major malaria vectors) populations from Burkina Faso and Mali (West Africa) are naturally infected by *Wolbachia* (Baldini et al., 2014; Gomes et al., 2017; Shaw et al., 2016). Notably, they showed a negative correlation between *Wolbachia* infection and *Plasmodium* development (Gomes et al., 2017; Shaw et al., 2016). Moreover, a very recent report suggests that other *Anopheles* species also are infected with *Wolbachia* (Jeffries, Golovko, et al., 2018; Jeffries, Lawrence, et al., 2018). These findings support the development of novel vector control strategies based on *Wolbachia–Anopheles* interactions. However, although *Wolbachia* naturally infects 40%–60% of arthropods (Duron et al., 2008; Zug & Hammerstein, 2012), infection of *Anopheles* species is still not well documented. Moreover, during the last decade, screens in many other malaria mosquito species worldwide (*n* = 38) did not bring any evidence of *Wolbachia* infection (Bourtzis et al., 2014; Hughes, Dodson, et al., 2014; Osei‐Poku, Han, Mbogo, & Jiggins, 2012).

In this study, we investigated the presence of *Wolbachia* in 25 *Anopheles* species in Gabon, Central Africa. We sampled mosquitoes across the country and in a variety of ecological settings, from deep rainforest to urban habitats. By using a molecular approach, we confirmed *Wolbachia* presence in 16 species, including all the major malaria vectors in Central Africa (*An. gambiae*,* An. coluzzii*,* Anopheles funestus, Anopheles nili* and *Anopheles moucheti*). The prevalence of *Wolbachia* infection was particularly high in *An. nili* and *An. moucheti*. Phylogenetic analysis revealed that all the infected mosquito species hosted *Wolbachia* bacteria belonging to the supergroup A or B (both exhibit high genetic diversity). Finally, we explored the co‐evolution between *Wolbachia* and *Anopheles*. The results have direct implications for the development of new and non‐insecticide‐based vector control strategies and open new directions for research on pathogen transmission and reproductive manipulation.

## 2 MATERIAL AND METHODS

### 2.1 Research and ethics statements

Mosquitoes were collected in Gabon under the research authorization AR0013/16/MESRS/CENAREST/CG/CST/CSAR and the national park entry authorization AE16008/PR/ANPN/SE/CS/AEPN. Mosquito sampling using the human‐landing catch (HLC) method was performed under the protocol 0031/2014/SG/CNE approved by the National Research Ethics Committee of Gabon.

### 2.2 Mosquito sampling and DNA extraction

**Figure 1:**
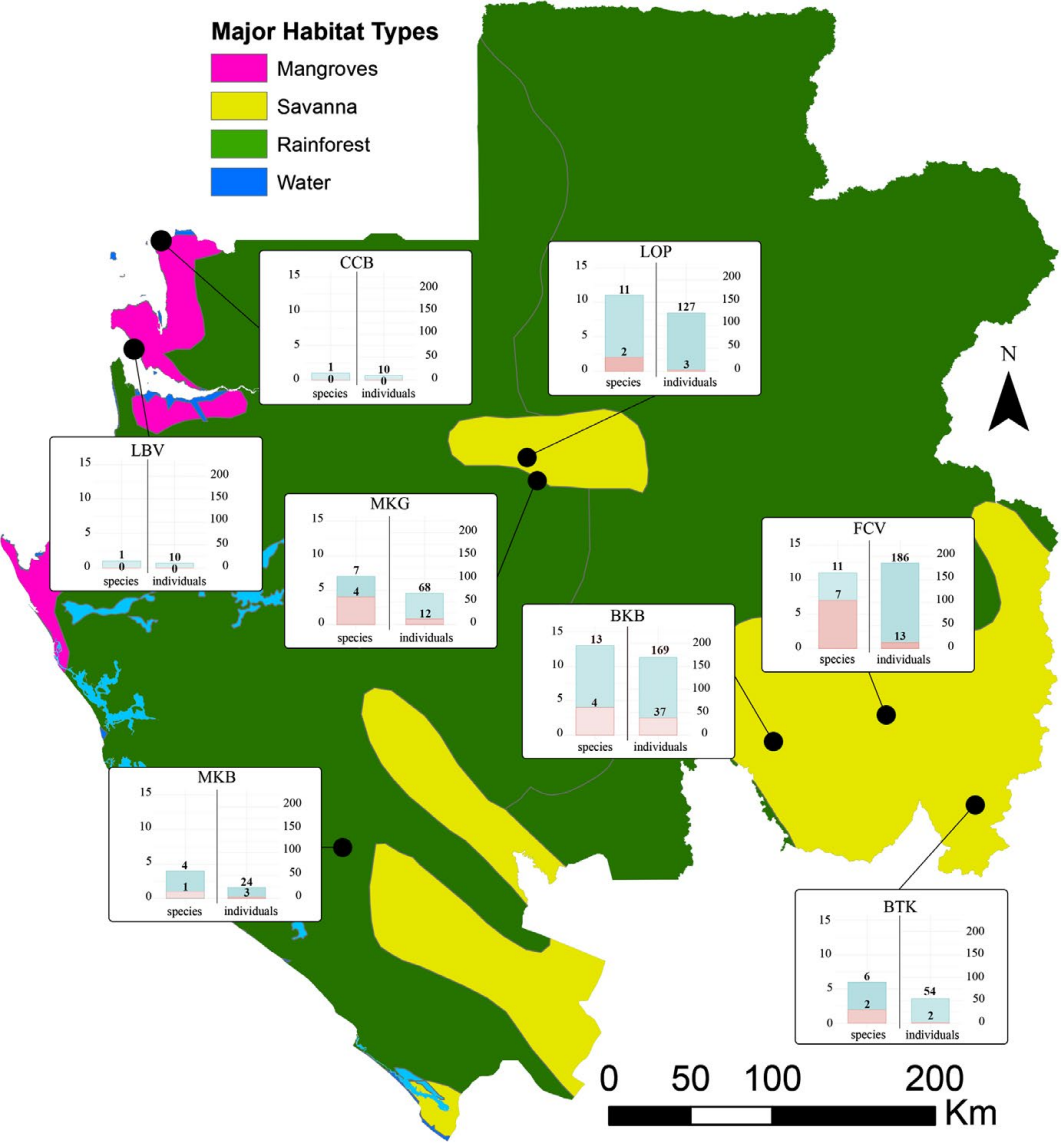
Sampling sites and *Wolbachia* infection prevalence. Map of Gabon showing the main African habitat types ((Olson et al., 2001), free ly available at http://maps.tnc.org/gis_data.html) and the villages where sampling took place (black dots). The map was drawn using ArcGIS Basic v.10. The prevalence of *Wolbachia* infection (number of infected *Anopheles* species and individuals) per site is presented in bar charts. The pink colour indicates positive species/individuals and blue the total number of species/individuals screened for *Wolbachia* infection at that site. BKB: Bakoumba; BTK: National Park of Plateaux Batékés; CCB: Cocobeach; FCV: Franceville; LBV: Libreville; LOP: Lopé; MKB: National Park of Moukalaba‐Doudou; MKG: Mikongo

Mosquitoes were collected in eight sites across Gabon, Central Africa, from 2012 to 2016 (Figure 1, Table 1, Appendix S1). These sites included sylvatic (national parks) and domestic habitats (villages and cities). Adult females were collected using Center for Disease Control () light traps, BioGents (BG) traps and HLC. Overall, CDC and BG were used in sylvatic and HLC in domestic sites (see Figure 1, Table S1). Collected specimens were taxonomically identified according to standard morphological features (Gillies & Coetzee, 1987; Gillies & de Meillon, 1968). Then, they were individually stored in 1.5 ml tubes at −20°C and sent to Centre International de Recherches Scientifiques de Franceville for molecular analysis. When possible, at least 30 mosquitoes (from 1 to 58) for each *Anopheles* species from different sites were selected for genomic analysis. Total genomic DNA was extracted from the whole body using the DNeasy Blood and Tissue Kit (Qiagen), according to the manufacturer's instructions. Genomic DNA was eluted in 100 μl of TE buffer. Specimens belonging to the *An. gambiae* complex, *An. funestus* group, *An. moucheti* complex and *An. nili* complex were molecularly identified using PCR‐based diagnostic protocols (Cohuet et al., 2003; Fanello, Santolamazza, & della Torre, 2002; Kengne et al., 2007; Kengne, Awono‐Ambene, Nkondjio, Simard, & Fontenille, 2003; Santolamazza et al., 2008).

**Table 1:** Summary of the *Anopheles* species screened in this study

Group/complex	Species	Malaria role	Infected	Tested	Infection (%)
gambiae	*An. gambiae*	H	5	44	11
	*An. coluzzii*	H	2	58	3
	*An. brunnipes*		0	1	0
	*An. cinctus*		0	2	0
moucheti	*An. moucheti*	H, P, A	30	42	71
	*An. nigeriensis*	h	1	27	4
	*An. "GAB−2"*		5	8	63
	*An. "GAB−3"*		1	1	100
	*An. gabonensis*	A	0	29	0
funestus	*An. funestus*	H	2	37	5
	*An. implexus*		1	26	4
	*An. jebudensis*		1	2	50
	*An. maculipalpis*	0	29	0	
nili	*An. nili*	H, A	11	19	58
	*An. carnevalei*	h, A	2	29	7
	*An. "GAB−1"*		0	19	0
	*An. hancocki*	h	1	41	2
	*An. theileri*	h	0	24	0
	*An. rodhesiensis*		0	4	0
coustani	*An. coustani*	h, A	2	35	6
	*An. paludis*	h, A	1	16	6
	*An. gr coustani*	h	0	51	0
	*An. squamosus*		0	32	0
	*An. marshallii*	h, P, A	2	42	5
	*An. vinckei*	P, A	3	30	10
Total		70	648		

Note

Malaria role: known role for each species in malaria transmission (Boundenga et al., 2016; Hamon & Mouchet, 1961; Makanga et al., 2016; Robert, Ayala, & Simard, 2017) in humans (H: major, h: secondary), primates (P), other animals (A) or unknown (blank).

### 2.3 *Wolbachia* screening and multilocus sequence typing analysis

*Wolbachia* infection in adult females was detected by nested PCR amplification of a *Wolbachia*‐specific *16S* rDNA fragment (~400 bp) using 2 μl of host genomic DNA, according to the protocol developed in Catteruccia's laboratory (Shaw et al., 2016). Amplification of this *16S* rDNA fragment in infected *Aedes albopictus* and *Culex pipiens* genomic DNA (data not shown) confirmed the performance of this nested PCR protocol to detect *Wolbachia* in many different mosquito species (Shaw et al., 2016). To detect potential contaminations, *Ae. albopictus* and *Culex quinquefasciatus* from Gabon were used as positive controls, and water and *Ae. aegypti* as negative controls. Moreover, PCR amplifications for each species were carried out independently and on different days. The amplicon size was checked on 1.5% agarose gels, and amplified *16S* rDNA fragments were sent to Genewiz (UK) for sequencing (forward and reverse) to confirm the presence of *Wolbachia*‐specific sequences. The DNA quality of all samples was confirmed by the successful amplification of a fragment (~800 bp) of the mitochondrial gene *COII* in all the *Anopheles* species under study (Ndo et al., 2010; Rahola et al., 2014). PCR products were run on 1.5% agarose gels, and *COII* fragments from 176 mosquito specimens of the 25 species were sequenced (forward and reverse) by Genewiz (UK) for the *Anopheles* phylogenetic studies. *Wolbachia*‐positive genomic DNA samples (2 μl/sample) were then genotyped by multilocus sequence typing (MLST) using three loci, *coxA *(~450 bp)* ftsZ *(~500 bp) and *fbpA *(~460 bp) (Baldo et al., 2006), and according to standard conditions (Baldo et al., 2006). If the three fragments could not be amplified, a newly developed nested PCR protocol was used. Specifically, after the first run with the standard primers, 2 μl of the obtained product was amplified again using internal primers specific for each gene: *coxA* (coxA_NF‐2: 5′‐TTTAACATGCGCGCAAAAGG‐3′; coxA_NR‐2: 5′‐TAAGCCCAACAGTGAACATATG‐3′), *ftsZ* (ftsZ_NF‐2: 5′‐ATGGGCGGTGGTACTGGAAC‐3′; ftsZ_NR‐2: 5′‐AGCACTAATTGCCCTATCTTCT‐3′) and *fbpA* (fbpA_NF‐1: 5′‐AGCTTAACTTCTGATCAAGCA‐3′; fbpA_NR‐1: 5′‐TTCTTTTTCCTGCAAAGCAAG‐3′). Cycling conditions for *coxA* and *ftsZ* were as follows: 94°C for 5 min, followed by 36 cycles at 94°C for 15 s, 55°C for 15 s and 72°C for 30 s, and a final extension step at 72°C for 10 min. For *fbpA*, they were: 94°C for 5 min followed by 36 cycles at 94°C for 30 s, 59°C for 45 s and 72°C for 90 s, and a final extension step at 72°C for 10 min. The resulting fragments (*coxA*, 357 bp; *fbpA*, 358 bp; and *ftsZ*, 424 bp) were sequenced bidirectionally by Genewiz. The new sequences obtained in this study were submitted to GenBank (Table S1). Unfortunately, the other three MLST genes (*gatB*, *wsp* and *hcpA*) could not be amplified, due to technical problems (i.e., multiple bands).

### 2.4 Phylogenetic and statistical analysis

All *Wolbachia* sequences for the *16S, coxA, fbpA* and *ftsZ* gene fragments and for *Anopheles* COII were manually corrected using *Geneious* R10 (Kearse et al., 2012). The resulting consensus sequences for each gene were aligned with sequences that represent the main known *Wolbachia* supergroups obtained from GenBank (see Table S1). Only unique haplotypes for each species were included in the analysis (haplotype was defined as a unique allelic profile for each examined locus). Inference of phylogenetic trees was performed using the maximum likelihood (ML) method and RAxML (Stamatakis, 2014) with a substitution model GTR + CAT (Stamatakis, 2006) and 1,000 bootstrapping replicates. Finally, all MLST *Wolbachi*a sequences were used to build phylogenetic trees using RAxML (GTR + CAT model, 1,000 bootstrapping replicates). Trees were visualized with iTOL v.3.4.3 (Letunic & Bork, 2007).

To quantify the accuracy of the observed *Wolbachia* infection prevalence, the influence of sample size on its estimation was assessed. For this, it was assumed that *Wolbachia* prevalence within a host species followed a beta binomial distribution (Zug & Hammerstein, 2012) yielding many species with a low or a high *Wolbachia* prevalence but few with an intermediate one. This allowed quantifying, for each sample size, the proportion of samples (over 1,000 realizations) that could yield an estimate that was not significantly different from the prevalence over the whole population with a *z* test and a significance threshold at 95%. As expected, sample size could be small for very low (<15%) or very high prevalence (>60%; 60 individuals are enough in 95% of cases for these extreme prevalence values), while it was much higher for intermediate prevalence values (up to 150 individuals for a prevalence value close to 50%).

All statistical analyses were performed using “R” v3.2.5 (R Development Core Team, http://cran.r-project.org/), with the addition of the “ggplot2” library (Wickham, 2009).

## 3 RESULTS

### 3.1 *Wolbachia* naturally infects a large number of *Anopheles* species from Gabon

In this study, we screened 648 mosquitoes from eight sites in Gabon (Figure 1, Table 1, Table S1). On the basis of their morphological traits (Gillies & Coetzee, 1987) and molecular analysis results (Cohuet et al., 2003; Kengne et al., 2007, 2003; Rahola et al., 2014; Santolamazza et al., 2008), we identified 25 *Anopheles* species (Appendix S1). Our sampling included all the species in which the presence of *Wolbachia* was previously investigated in Africa (*An. gambiae*,* An. coluzzii*,* An. funestus* and *Anopheles coustani*), with the exception of *An. arabiensis* that is absent in Gabon (Table 1) (Makanga et al., 2016). By PCR amplification of a *16S* rRNA fragment (Shaw et al., 2016), we found 70 *Wolbachia*‐positive specimens that belonged to 16 different *Anopheles* species, distributed throughout the country (Figure 1, Table S1). When considering only species with more than 10 screened individuals, we observed that Wolbachia infection was commonly lower than 15% (11/13), as observed in other arthropods (Duron et al., 2008; Zug & Hammerstein, 2012). On the other hand, two species, and moreover major malaria vectors, *An. moucheti* and *An. nili,* exhibited more than 50% of *Wolbachia* infection (Table 1), as previously reported in other mosquito species where prevalence can be very high (Dumas et al., 2013; Duron et al., 2005).

### 3.2 *Wolbachia* is maternally inherited in *An. moucheti*

Although *Wolbachia* is mainly maternally transmitted (Werren et al., 2008), horizontal transmission may occasionally occur in natural conditions (Ahmed, De Barro, Ren, Greeff, & Qiu, 2013; Li et al., 2017; Werren, Zhang, & Guo, 1995). To confirm the maternal transmission in the infected mosquito species, we focused on *An. moucheti* for logistic reasons (i.e., highest *Wolbachia* prevalence and ease of sampling). Although no laboratory *An. moucheti* strain is currently available, we obtained eggs from six *Wolbachia*‐infected females. In total, we analysed the infectious status of 79 progeny by PCR amplification of the same *16S* rRNA fragment (Shaw et al., 2016) (Table S3) and found that 70 were infected, with an average maternal transmission frequency of 97.54% (range: 90%–100%).

### 3.3 Naturally occurring *Wolbachia* strains in *Anopheles* reveal high genetic diversity

**Figure 2:**
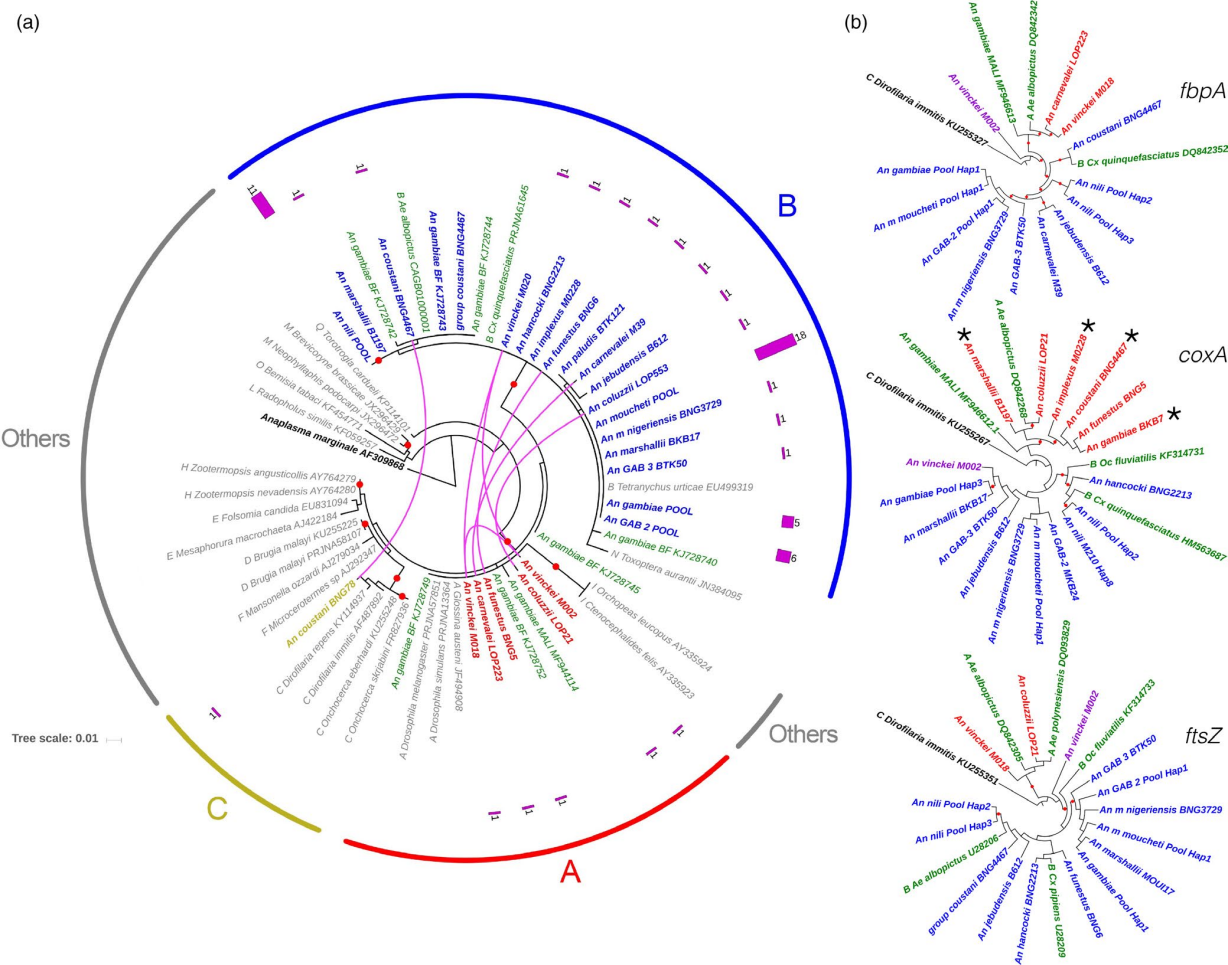
Circular phylograms of the *Wolbachia* strains isolated in the 16 *Anopheles* species. The phylogenetic trees were built with RAxML (Stamatakis, 2014). The names of the *Anopheles* species from which the *Wolbachia*‐specific sequences were isolated in this study are shown in blue (positive for *Wolbachia* supergroup B), red (positive for supergroup A) and brown (positive for supergroup C), while the names of mosquitoes species (*Diptera*) from which the previously published *Wolbachia* sequences were isolated are in green. Other *Wolbachia* strains sequences (“others,” in grey) were obtained directly from gene sequence repository ncbi (https://www.ncbi.nlm.nih.gov/). Red dots show branches supporting a bootstrap >70% from 1,000 replicates. (a) Circular phylogenetic tree using the *Wolbachia*‐specific *16S* rRNA fragment and *Anaplasma marginale* as outgroup. Different *Wolbachia* strains found in the same *Anopheles* species are connected by pink lines. The pink bar charts indicate the number of identical *Wolbachia* haplotypes found in each species. Scale bar corresponds to nucleotide substitutions per site. (b) Circular phylogenetic trees based on the *coxA, fbpA and ftsZ* fragment sequences using *Dirofilaria immitis* (supergroup C) as outgroup. Specimens with a different supergroup assignation than *16S* are marked with asterisks. Only, *Anopheles vinckei* M002 (purple) oscillated between groups B and A across the four genes

By sequence analysis of the *16S* rRNA fragment PCR amplified from each *Anopheles* sample (Table 1), we could assign the *Wolbachia* strains to three pre‐existing supergroups: A (*n* = 5), B (*n* = 64) and C (*n* = 1; Figure 2). Specifically, we detected supergroup B *Wolbachia* in 64 mosquitoes belonging to all 16 infected *Anopheles* species. We found supergroup A *Wolbachia* in five individuals from four species (*An. funestus, An. coluzzii, Anopheles vinckei* and *Anopheles carnevalei*), thus providing examples of multiple infections, as previously observed in *Ae. albopictus* (Sinkins, Braig, & Oneill, 1995) (Figure 2). None of the mosquitoes examined was co‐infected by *Wolbachia* strains belonging, for instance, to the supergroups A and B. Moreover, we confirmed that the *Wolbachia* strains previously identified in *An. gambiae *s.l. from Burkina Faso and Mali are included in the supergroups A and B (Baldini et al., 2014; Gomes et al., 2017). Finally, we found that one *An. coustani* individual was infected by a *Wolbachia* strain from supergroup C that is known to infect only filarial worms. Therefore, we investigated the presence of filarial nematode DNA in the mosquito by PCR amplification and sequencing of a fragment of the *COI* filarial gene (Casiraghi, Anderson, Bandi, Bazzocchi, & Genchi, 2001), followed by phylogenetic analysis with RAxML. Our results confirmed the presence of *Dirofilaria immitis* in this specimen (Figure S1). This canine filarial parasite hosts *Wolbachia* and is transmitted by many mosquitoes, including *Anopheles* (Simon et al., 2012). Therefore, it is not surprising to find an *An. coustani* specimen infected by this filarial nematode.

**Figure 3:**
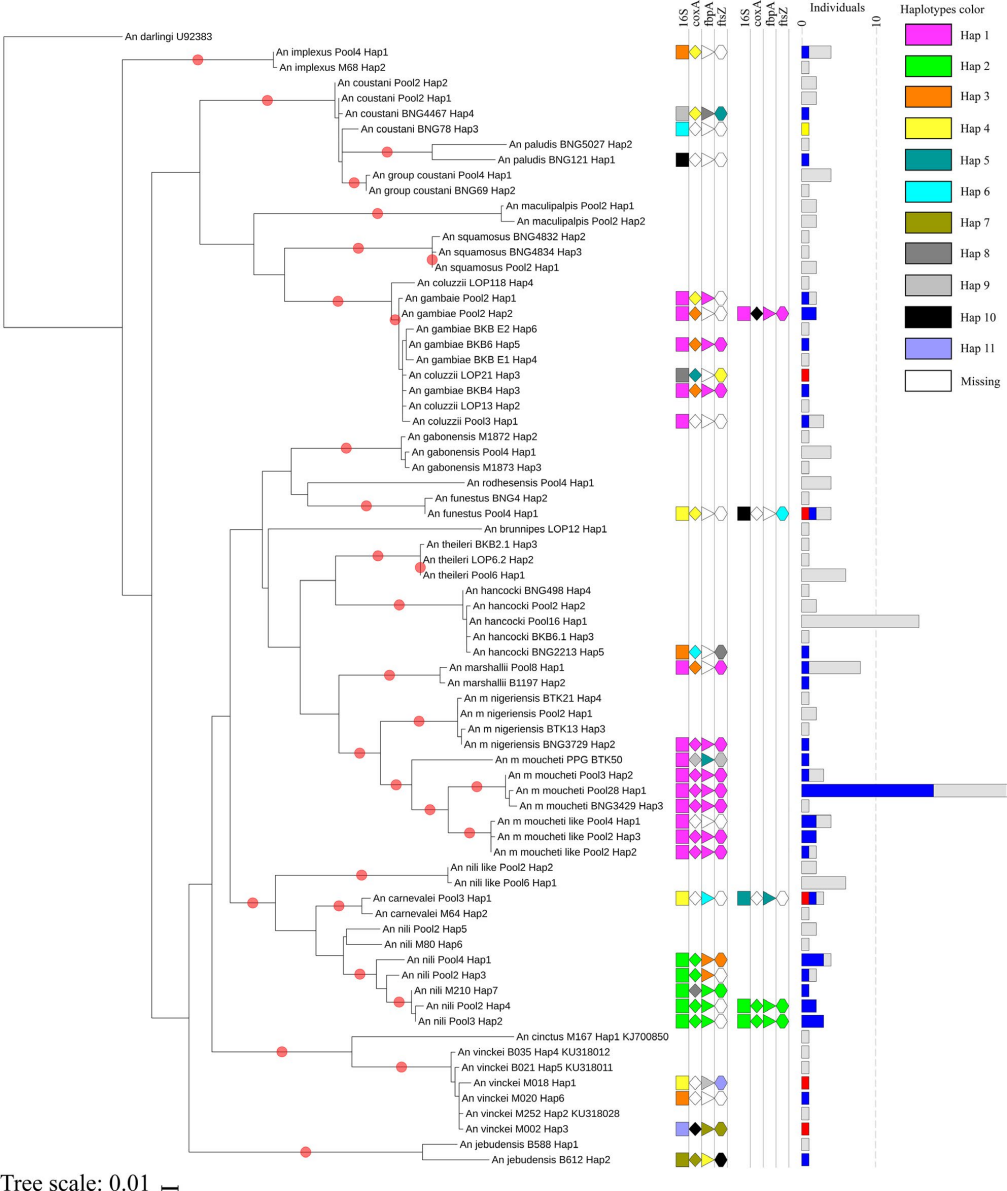
Maximum likelihood phylogeny of the 25 *Anopheles* species under study and *Wolbachia* haplotypes. The tree was inferred with RAxML (Stamatakis, 2014) using the sequences of the *COII* fragment from 176 *Anopheles* specimens belonging to the 25 species under study and rooted with *Anopheles darlingi* as outgroup (New World mosquito, diverged 100 Myr ago (Neafsey et al., 2015)). Red dots in branches represent bootstrap values >70% from 1,000 replicates. The shape of each field column represents the *16S* (rectangle), *coxA* (rhombus), *fbpA* (triangle) and *ftsZ* (hexagon) genes. The different *Wolbachia* gene haplotypes (i.e., unique allelic profiles) are indicated with colour codes (all pink = the newly identified *w*Anmo strain). The bar chart size indicates the number of individuals of the same species with the same haplotype, and the colour represents their infection status: grey, noninfected; blue, infected by the *Wolbachia* supergroup B; red, infected by supergroup A; brown, infected by supergroup C

To expand our knowledge on the *Wolbachia* strains that infect natural *Anopheles* populations, we PCR amplified, sequenced and analysed fragments from three conserved *Wolbachia* genes (*coxA, fbpA* and *ftsZ*) that are commonly used for strain typing and evolutionary studies (Baldo et al., 2006) (Figure 2). We used a new nested PCR protocol (see section 22) for samples that could not be genotyped using the classical MLST primers (Table S1). Our phylogenetic analyses confirmed the *16S* results, assigning most of the species to supergroups A and B. Few samples (asterisks in Figure 2, gene *coxA*) showed some incongruence relative to the *16S* results. They suggest signals of recent recombination between the supergroups A and B, as previously demonstrated (Baldo et al., 2006). Detailed sequence analysis revealed that mosquito species belonging to the same group or complex (i.e., *An. moucheti* and *An. gambiae*) displayed a common *Wolbachia* haplotype (defined here as a unique allelic profile; Figures 2 and 3). Conversely, some species with lower prevalence (i.e., *An. coluzzii, An. marshallii, An. vinckei* or *An. funestus*) displayed a variety of haplotypes. The case of *An. vinckei* was particularly interesting because the three infected specimens displayed different haplotypes for the analysed *Wolbachia* genes. Moreover, one specimen (*An. vinckei* M002, Figure 2) was infected by a completely different *Wolbachia* strain. Overall, the *Wolbachia* haplotypes identified in this study were different from the allelic profiles of the previously annotated *Wolbachia* strains or of the strain that infects *An. gambiae* in Burkina Faso and Mali (Baldini et al., 2014; Gomes et al., 2017) (Figures 2 and 3). Within supergroup B, we could easily distinguish at least two strains. The strain infecting *An*. *moucheti* (*w*Anmo), which showed no variation across localities, was similar to the one identified in *An. gambiae* (in our study) or *Anopheles marshallii*, while the strain infecting *An*. *nili* (*w*Anni), which evidenced strains variation even in the same locality, was more closely related to those found in other mosquito species, such as *Ae. albopictus* or *Cx. quinquefasciatus* (Figures 2 and 3). Conversely, the other haplotypes were associated with one specific host.

### 3.4 *Wolbachia* independently evolves in malaria‐transmitting mosquitoes

As *Wolbachia* is mainly a maternally inherited bacterium, the host mitochondrial DNA (mtDNA) is a suitable marker to study its evolutionary history in *Anopheles* (Richardson et al., 2012). Analysis of *COII* sequences from 176 specimens belonging to the 25 *Anopheles* species collected in Gabon provided the most exhaustive phylogenetic tree of *Anopheles* in Central Africa (Figure 3). This analysis highlighted the independent acquisition and apparent loss of *Wolbachia* across the different *Anopheles* species clades. Moreover, the genetic distances of *Wolbachia* strains and their *Anopheles* host were not correlated (Mantel test, *p* > 0.05; Figure S2). Nevertheless, mosquitoes from the *An. moucheti* complex, and therefore genetically very close, shared the same *Wolbachia* supergroup and haplotypes (Figure 3 and Figure S2). Finally, we investigated how *Wolbachia* evolved within each *Anopheles* species (Charlat et al., 2009). Our results revealed that *Wolbachia*‐infected and noninfected mosquitoes shared the same mtDNA haplotype (Figure 3), indicating that infection status and host haplotypes are not associated.

## 4 DISCUSSION

The present study provides three key findings. First, the genus *Anopheles* includes a large number of species that are naturally infected by *Wolbachia* (16/25), with high infection prevalence among major malaria vectors. Second, *Anopheles‐*infecting *Wolbachia* bacteria show high genetic diversity, with similar haplotypes detected in different *Anopheles* species. Third, the independent evolution of *Wolbachia* and *Anopheles* might be interpreted as multiple acquisition events with horizontal transmission. The large diversity of *Wolbachia* strains that infect many natural *Anopheles* populations could represent a major opportunity for reducing pathogen transmission and/or for reproductive manipulation in *Anopheles* with the aim of decreasing malaria burden in Africa.

During the last decades, the scientific community has evidenced an interest to find new ways to use *Wolbachia* for fighting vector‐borne diseases (Bourtzis et al., 2014; Hoffmann et al., 2015; Iturbe‐Ormaetxe et al., 2011; McGraw & O'Neill, 2013). In arthropods, *Wolbachia* infection is very common, including among *Culex* and *Aedes* mosquitoes. Conversely, the genus *Anopheles* revealed no infection to the bacteria. Until recently, *Wolbachia* infections were mainly limited to species within the gambiae complex (Baldini et al., 2014; Gomes et al., 2017) and few other species (Baldini et al., 2018; Jeffries, Golovko, et al., 2018; Jeffries, Lawrence, et al., 2018; Niang et al., 2018). Several hypotheses can be put forward to explain this. First, low infection prevalence or local variations could have hindered the discovery of *Wolbachia* infections, independently of the sampling effort. In our study, most *Anopheles* species exhibited a prevalence lower than 15% (Table 1). This pattern is common in many other arthropods (Duron et al., 2008; Zug & Hammerstein, 2012), and it is usually associated with a weak manipulation of the host reproduction and/or imperfect maternal transmission (Engelstadter & Hurst, 2009). In general, our sampling effort was higher than in previous studies (*n* < 30) (Bourtzis et al., 2014; Osei‐Poku et al., 2012), and this could explain why we found more infected species. Our statistical analysis showed that a sample size of 60 individuals per species is needed to quantify correct prevalence rates lower than 15%, with a probability of 95% (Figure S3). Moreover, local frequency variations among populations could also hinder the detection of *Wolbachia* infections (Dumas et al., 2013). For instance, we sampled *An. coluzzii* in three different sites, but we only found *Wolbachia*‐infected mosquitoes at La Lopé (Figure 1, Table S1). Therefore, sampling in different localities and in different seasons might improve detection rates. Second, it could be difficult to detect low‐density *Wolbachia* infections in *Anopheles* with the routinely used molecular tools, as previously reported for other arthropods (Arthofer, Riegler, Avtzis, & Stauffer, 2009; Augustinos et al., 2011) and recently in *An. gambiae* (Gomes et al., 2017). Our results indicate that conventional PCR amplification (*wsp*‐targeting primers (Baldo et al., 2006)) analysis allowed the detection of *Wolbachia* infection only in 6 of the 16 species (*An. moucheti*,* Anopheles m. nigeriensis, An. “GAB‐3,” An. nili, Anopheles jebudensis* and *An. vinckei*) under study, presumably because of the high *Wolbachia* density. Moreover, some *Anopheles* species with high *Wolbachia* infection rates, such as *An. moucheti* or *An. nili*, were never screened before.

Our work revealed that *Anopheles* species are infected by different *Wolbachia* strains. Although t previous studies reported *Wolbachia* infection in *Anopheles* (Baldini et al., 2014; Gomes et al., 2017; Jeffries, Golovko, et al., 2018; Jeffries, Lawrence, et al., 2018; Niang et al., 2018; Shaw et al., 2016), there exist the doubt if they are real infections (Chrostek & Gerth, 2018). The *Wolbachia* sequences found in our specimens were genetically close to those found in other Diptera, and no signal of extensive divergence was detected (Figures 2 and 3). Therefore, there is no risk that horizontal gene transfer (resulting in the insertion of *Wolbachia* genes within the mosquito genome) or parasitism (e.g., by filarial nematodes) could explain the detection of *Wolbachia* genes in our infected mosquitoes without maternal transmission. Moreover, the analysis of *An. moucheti* F1 progeny confirms, at least in this species, that no other biological *Wolbachia* contamination was present in our analysis. In conclusion, our data suggest that *Wolbachia* is naturally present in the *Anopheles* species of Central Africa analysed in our study, and that it is maternally inherited in *An. moucheti* (Table S2). In this sense and besides the challenge to rear *An. moucheti* under insectary conditions, this mosquito should be considered as potential model species to study the reproductive phenotypes of *Wolbachia* and its effect in *Plasmodium* infections.

In Central African *Anopheles*, *Wolbachia* acquisition seems to be independent of the host phylogeny (Figures 2 and 3). Our results revealed that the genetic distances between *Wolbachia* and *Anopheles* are not positively correlated (Mantel test, *p* > 0.05; Figure S2). The lack of correlation could lead to think that *Wolbachia* and the host lineage evolved independently. The different larval ecology of these species suggests other ways of lateral transfer (e.g., during nectar feeding (Li et al., 2017)). On the other hand, we found that species belonging to *An. moucheti* complex shared related *Wolbachia* strains (Figure 3). Permeable reproductive barriers among members of the same complex could facilitate the intermittent movement of the bacterium (Pombi et al., 2017). Interestingly, although they share similar *Wolbachia* strains, sibling species showed different infection prevalence. Indeed, *An. carnevalei* and *An. m. nigeriensis* exhibited frequencies lower than 15%, whereas *An. nili* and *An. moucheti*, their respective counterparts and the most important malaria vectors in their complex, displayed frequencies higher than 50% (Table 1). Moreover, our *An. gambiae* and *An. coluzzii* populations were infected by different *Wolbachia* strains than those detected in Burkina Faso and Mali. Similarly, in mosquitoes (Dumas et al., 2013) and ants (Tsutsui, Kauppinen, Oyafuso, & Grosberg, 2003), the same species is infected by different *Wolbachia* strains according to the region. The availability of whole‐genome sequences for *Wolbachia* strains (Gerth, Gansauge, Weigert, & Bleidorn, 2014) will enlighten the intricate phylogenetic relationships among the different strains in *Anopheles*.

## 5 CONCLUSIONS

*Wolbachia* has emerged as a biological tool for controlling vector‐borne diseases (Hoffmann et al., 2011; Schmidt et al., 2017). In this study, we demonstrated the natural presence of this endosymbiont bacterium in a large number of *Anopheles* species, including the five major malaria vectors in Central Africa. Previously, it has been shown that *Wolbachia* ability to interfere with pathogen transmission depends on the bacterium strain (Blagrove, Arias‐Goeta, Failloux, & Sinkins, 2012; Kambris et al., 2010; Walker et al., 2011). Therefore, our results offer the opportunity to determine whether the different *Anopheles*‐infecting *Wolbachia* strains affect *Plasmodium* transmission and/or *Anopheles* reproduction. Indeed, three major vectors of human and nonhuman malaria (*An. moucheti*, *An. nili* and *An. vinckei*) were infected by *Wolbachia* (Makanga et al., 2016; Paupy et al., 2013). Therefore, we could investigate both *Wolbachia‐*mediated decreases (Hughes, Rivero, & Rasgon, 2014; Zele et al., 2014) and increases (Shaw et al., 2016) in susceptibility of these natural vectors to *Plasmodium*. Moreover, the strongest effect on suppression of pathogen transmission or reproductive manipulation has been observed in *Wolbachia* transinfections (Bian et al., 2013; Bian, Xu, Lu, Xie, & Xi, 2010; Blagrove et al., 2012; Hughes et al., 2011; Joubert et al., 2016; Moreira et al., 2009; Walker et al., 2011). Therefore, the availability of *Wolbachia* strains that infect natural *Anopheles* populations offers promising opportunities for experimental and theoretical studies in *Anopheles*, and also in other mosquito families that are vectors of other diseases, including *Ae. aegypti* and *Ae. albopictus*. In conclusion, our findings are merely the “tip of the iceberg” of *Wolbachia* research in *Anopheles*. The selection of suitable phenotypes for suppressing *Plasmodium* transmission and/or manipulating *Anopheles* reproduction could greatly participate to reduce the malaria burden across the world.

## CONFLICT OF INTEREST

None declared.

## Data Availability

Data for this study are available at the Dryad digital Repository: https://doi.org/10.5061/dryad.sn81548 (Ayala et al., 2019). DNA sequences of *Wolbachia* and *Anopheles* recovered in this study and of those used as references for phylogenetic analyses are submitted at Genbank (MK755460–MK755837).

## References

[eva12804-bib-0001] Ahmed, M. Z. , De Barro, P. J. , Ren, S. X. , Greeff, J. M. , & Qiu, B. L. (2013). Evidence for horizontal transmission of secondary endosymbionts in the Bemisia tabaci cryptic species complex. PLoS ONE, 8(1), e53084 10.1371/journal.pone.0053084 23308142 PMC3538644

[eva12804-bib-0002] Arthofer, W. , Riegler, M. , Avtzis, D. N. , & Stauffer, C. (2009). Evidence for low‐titre infections in insect symbiosis: Wolbachia in the bark beetle Pityogenes chalcographus (Coleoptera, Scolytinae). Environmental Microbiology, 11(8), 1923–1933. 10.1111/j.1462-2920.2009.01914.x 19383035

[eva12804-bib-0003] Augustinos, A. A. , Santos‐Garcia, D. , Dionyssopoulou, E. , Moreira, M. , Papapanagiotou, A. , Scarvelakis, M. , … Bourtzis, K. (2011). Detection and characterization of Wolbachia Infections in Natural Populations of Aphids: Is the hidden diversity fully unraveled? PLoS ONE, 6(12), e28695 10.1371/journal.pone.0028695 22174869 PMC3236762

[eva12804-bib-0004] Ayala, D. , Akone‐Ella, O. , Rahola, N. , Kengne, P. , Ngangue, M. F. , Mezeme, F. , … Paupy, C. (2019). Natural Wolbachia infections are common in the major malaria vectors in Central Africa. Evolutionary Applications. Dryad digital Repository; 10.5061/dryad.sn81548 PMC6708434 31462916

[eva12804-bib-0005] Baldini, F. , Rouge, J. , Kreppel, K. , Mkandawile, G. , Mapua, S. A. , Sikulu‐Lord, M. , … Okumu, F. O. (2018). First report of natural Wolbachia infection in the malaria mosquito *Anopheles arabiensis* in Tanzania. Parasit Vectors, 11(1), 635 10.1186/s13071-018-3249-y 30545384 PMC6293665

[eva12804-bib-0006] Baldini, F. , Segata, N. , Pompon, J. , Marcenac, P. , Shaw, W. R. , Dabire, R. K. , … Catteruccia, F. (2014). Evidence of natural Wolbachia infections in field populations of Anopheles gambiae. Nature Communications, 5, 3985 10.1038/ncomms4985 PMC4059924 24905191

[eva12804-bib-0007] Baldo, L. , Dunning Hotopp, J. C. , Jolley, K. A. , Bordenstein, S. R. , Biber, S. A. , Choudhury, R. R. , … Werren, J. H. (2006). Multilocus sequence typing system for the endosymbiont Wolbachia pipientis. Applied and Environmental Microbiology, 72(11), 7098–7110. 10.1128/aem.00731-06 16936055 PMC1636189

[eva12804-bib-0008] Bhatt, S. , Weiss, D. J. , Cameron, E. , Bisanzio, D. , Mappin, B. , Dalrymple, U. , & Gething, P. W. (2015). The effect of malaria control on Plasmodium falciparum in Africa between 2000 and 2015. Nature, 526(7572), 207–211. 10.1038/nature15535 26375008 PMC4820050

[eva12804-bib-0009] Bian, G. , Joshi, D. , Dong, Y. , Lu, P. , Zhou, G. , Pan, X. , … Xi, Z. (2013). Wolbachia invades *Anopheles stephensi* populations and induces refractoriness to plasmodium infection. Science, 340(6133), 748–751. 10.1126/science.1236192 23661760

[eva12804-bib-0010] Bian, G. , Xu, Y. , Lu, P. , Xie, Y. , & Xi, Z. (2010). The endosymbiotic bacterium Wolbachia induces resistance to dengue virus in *Aedes aegypti* . PLoS Path, 6(4), e1000833 10.1371/journal.ppat.1000833 PMC2848556 20368968

[eva12804-bib-0011] Blagrove, M. S. C. , Arias‐Goeta, C. , Failloux, A. B. , & Sinkins, S. P. (2012). Wolbachia strain wMel induces cytoplasmic incompatibility and blocks dengue transmission in *Aedes albopictus* . Proceedings of the National Academy of Sciences of the United States of America, 109(1), 255–260. 10.1073/pnas.1112021108 22123944 PMC3252941

[eva12804-bib-0012] Boundenga, L. , Makanga, B. , Ollomo, B. , Gilabert, A. , Rougeron, V. , Mve‐Ondo, B. , … Paupy, C. (2016). Haemosporidian parasites of antelopes and other vertebrates from Gabon, Central Africa. PLoS ONE, 11(2), e0148958 10.1371/journal.pone.0148958 26863304 PMC4749209

[eva12804-bib-0013] Bourtzis, K. , Dobson, S. L. , Xi, Z. Y. , Rasgon, J. L. , Calvitti, M. , Moreira, L. A. , … Gilles, J. R. L. (2014). Harnessing mosquito‐Wolbachia symbiosis for vector and disease control. Acta Tropica, 132, S150–S163. 10.1016/j.actatropica.2013.11.004 24252486

[eva12804-bib-0014] Casiraghi, M. , Anderson, T. J. C. , Bandi, C. , Bazzocchi, C. , & Genchi, C. (2001). A phylogenetic analysis of filarial nematodes: Comparison with the phylogeny of Wolbachia endosymbionts. Parasitology, 122, 93–103. 10.1017/s0031182000007149 11197770

[eva12804-bib-0015] Charlat, S. , Duplouy, A. , Hornett, E. A. , Dyson, E. A. , Davies, N. , Roderick, G. K. , … Hurst, G. D. (2009). The joint evolutionary histories of Wolbachia and mitochondria in Hypolimnas bolina. BMC Evolutionary Biology, 9(1), 64 10.1186/1471-2148-9-64 19317891 PMC2669805

[eva12804-bib-0016] Chrostek, E. , & Gerth, M. (2018). Is anopheles gambiae a natural host of Wolbachia? bioRxiv. 10.1101/491449 PMC6561020 31186318

[eva12804-bib-0017] Cohuet, A. , Simard, F. , Toto, J. C. , Kengne, P. , Coetzee, M. , & Fontenille, D. (2003). Species identification within the *Anopheles funestus* group of malaria vectors in Cameroon and evidence for a new species. American Journal of Tropical Medicine and Hygiene, 69(2), 200–205. 13677376

[eva12804-bib-0018] Dodson, B. L. , Hughes, G. L. , Paul, O. , Matacchiero, A. C. , Kramer, L. D. , & Rasgon, J. L. (2014). Wolbachia enhances West Nile Virus (WNV) infection in the mosquito Culex tarsalis. Plos Neglected Tropical Diseases, 8(7), e2965 10.1371/journal.pntd.0002965 25010200 PMC4091933

[eva12804-bib-0019] Dumas, E. , Atyame, C. M. , Milesi, P. , Fonseca, D. M. , Shaikevich, E. V. , Unal, S. , … Duron, O. (2013). Population structure of Wolbachia and cytoplasmic introgression in a complex of mosquito species. BMC Evolutionary Biology, 13, 181 10.1186/1471-2148-13-181 24006922 PMC3846486

[eva12804-bib-0020] Duron, O. , Bouchon, D. , Boutin, S. , Bellamy, L. , Zhou, L. , Engelstaedter, J. , & Hurst, G. D. (2008). The diversity of reproductive parasites among arthropods: Wolbachia do not walk alone. BMC Biology, 6, 1–12. 10.1186/1741-7007-6-27 18577218 PMC2492848

[eva12804-bib-0021] Duron, O. , Lagnel, J. , Raymond, M. , Bourtzis, K. , Fort, P. , & Weill, M. (2005). Transposable element polymorphism of Wolbachia in the mosquito *Culex pipiens*: Evidence of genetic diversity, superinfection and recombination. Molecular Ecology, 14, 1561–1573. 10.1111/j.1365-294X.2005.02495.x 15813794

[eva12804-bib-0022] Engelstadter, J. , & Hurst, G. D. D. (2009). The ecology and evolution of microbes that manipulate host reproduction. Annual Review of Ecology Evolution and Systematics, 40, 127–149. 10.1146/annurev.ecolsys.110308.120206

[eva12804-bib-0023] Fanello, C. , Santolamazza, F. , & della Torre, A. (2002). Simultaneous identification of species and molecular forms of the *Anopheles gambiae* complex by PCR‐RFLP. Medical and Veterinary Entomology, 16(4), 461–464. 12510902 10.1046/j.1365-2915.2002.00393.x

[eva12804-bib-0024] Gerth, M. , Gansauge, M. T. , Weigert, A. , & Bleidorn, C. (2014). Phylogenomic analyses uncover origin and spread of the Wolbachia pandemic. Nature Communications, 5, 1–7. 10.1038/ncomms6117 25283608

[eva12804-bib-0025] Gillies, M. T. , & Coetzee, M. C. (1987). A supplement to the anophelinae of Africa south of the Sahara (Afrotropical region). Johannesburg, South Africa: The South African Institute for Medical Research.

[eva12804-bib-0026] Gillies, M. T. , & de Meillon, B. (1968). The anophelinae of Africa, south of the Sahara, Vol. 54 Johannesburg, South Africa: The South African Institute for Medical Research.

[eva12804-bib-0027] Gomes, F. M. , Hixson, B. L. , Tyner, M. D. W. , Ramirez, J. L. , Canepa, G. E. , Silva, T. , … Barillas‐Mury, C. (2017). Effect of naturally occurring *Wolbachia* in A*nopheles gambiae *s.l. mosquitoes from Mali on *Plasmodium falciparum* malaria transmission. PNAS, 114(47), 12566–12571. 10.1073/pnas.1716181114 29114059 PMC5703331

[eva12804-bib-0028] Griffi, J. T. , Bhatt, S. , Sinka, M. E. , Gething, P. W. , Lynch, M. , Patouillard, E. , … Ghani, A. C. (2016). Potential for reduction of burden and local elimination of malaria by reducing Plasmodium falciparum malaria transmission: A mathematical modelling study. Lancet Infectious Diseases, 16(4), 465–472. 10.1016/s1473-3099(15)00423-5 26809816 PMC5206792

[eva12804-bib-0029] Hamon, J. , & Mouchet, J. (1961). Secondary vectors of human malaria in Africa. Medecine Tropicale, 21, 643–660. 13904211

[eva12804-bib-0030] Hoffmann, A. A. , Montgomery, B. L. , Popovici, J. , Iturbe‐Ormaetxe, I. , Johnson, P. H. , Muzzi, F. , … O'Neill, S. L. (2011). Successful establishment of Wolbachia in Aedes populations to suppress dengue transmission. Nature, 476(7361), 454–U107. 10.1038/nature10356 21866160

[eva12804-bib-0031] Hoffmann, A. A. , Ross, P. A. , & Rasic, G. (2015). Wolbachia strains for disease control: Ecological and evolutionary considerations. Evolutionary Applications, 8(8), 751–768. 10.1111/eva.12286 26366194 PMC4561566

[eva12804-bib-0032] Hughes, G. L. , Dodson, B. L. , Johnson, R. M. , Murdock, C. C. , Tsujimoto, H. , Suzuki, Y. , … Rasgon, J. L. (2014). Native microbiome impedes vertical transmission of Wolbachia in Anopheles mosquitoes. Proceedings of the National Academy of Sciences, 111(34), 12498–12503. 10.1073/pnas.1408888111 PMC4151774 25114252

[eva12804-bib-0033] Hughes, G. L. , Koga, R. , Xue, P. , Fukatsu, T. , & Rasgon, J. L. (2011). Wolbachia infections are virulent and inhibit the human malaria parasite *Plasmodium falciparum* in *Anopheles gambiae* . PLoS Path, 7(5), e1002043 10.1371/journal.ppat.1002043 PMC3098226 21625582

[eva12804-bib-0034] Hughes, G. L. , Rivero, A. , & Rasgon, J. L. (2014). Wolbachia can enhance plasmodium infection in mosquitoes: Implications for malaria control? PLoS Path, 10(9), e1004182 10.1371/journal.ppat.1004182 PMC4154766 25187984

[eva12804-bib-0035] Hughes, G. L. , Vega‐Rodriguez, J. , Xue, P. , & Rasgon, J. L. (2012). Wolbachia strain wAlbB enhances infection by the rodent malaria parasite *Plasmodium berghei* in *Anopheles gambiae* mosquitoes. Applied and Environmental Microbiology, 78(5), 1491–1495. 10.1128/aem.06751-11 22210220 PMC3294472

[eva12804-bib-0036] Iturbe‐Ormaetxe, I. , Walker, T. , & Neill, S. L. O. (2011). Wolbachia and the biological control of mosquito‐borne disease. EMBO Reports, 12(6), 508–518. 10.1038/embor.2011.84 21546911 PMC3128286

[eva12804-bib-0037] Jeffries, C. L. , Golovko, G. , Kristan, M. , Orsborne, J. , Spence, K. , Hurn, E. , & Walker, T. (2018). Novel Wolbachia strains in Anopheles malaria vectors from Sub‐Saharan Africa. bioRxiv 10.1101/338434 PMC6234743 30483601

[eva12804-bib-0038] Jeffries, C. L. , Lawrence, G. G. , Golovko, G. , Kristan, M. , Orsborne, J. , Spence, K. , … Walker, T. (2018). Novel Wolbachia strains in Anopheles malaria vectors from Sub‐Saharan Africa. Wellcome Open Res, 3, 113 10.12688/wellcomeopenres.14765.2 30483601 PMC6234743

[eva12804-bib-0039] Joubert, D. A. , Walker, T. , Carrington, L. B. , De Bruyne, J. T. , Kien, D. H. , Hoang Nle, T. , … O'Neill, S. L. (2016). Establishment of a wolbachia superinfection in *Aedes aegypti* mosquitoes as a potential approach for future resistance management. PLoS Path, 12(2), e1005434 10.1371/journal.ppat.1005434 PMC4758728 26891349

[eva12804-bib-0040] Kambris, Z. , Blagborough, A. M. , Pinto, S. B. , Blagrove, M. S. C. , Godfray, H. C. J. , Sinden, R. E. , & Sinkins, S. P. (2010). Wolbachia stimulates immune gene expression and inhibits plasmodium development in *Anopheles gambiae* . PLoS Path, 6(10), e1001143 10.1371/journal.ppat.1001143 PMC2951381 20949079

[eva12804-bib-0041] Kambris, Z. , Cook, P. E. , Phuc, H. K. , & Sinkins, S. P. (2009). Immune activation by life‐shortening wolbachia and reduced filarial competence in mosquitoes. Science, 326(5949), 134–136. 10.1126/science.1177531 19797660 PMC2867033

[eva12804-bib-0042] Kearse, M. , Moir, R. , Wilson, A. , Stones‐Havas, S. , Cheung, M. , Sturrock, S. , … Drummond, A. (2012). Geneious Basic: An integrated and extendable desktop software platform for the organization and analysis of sequence data. Bioinformatics, 28(12), 1647–1649. 10.1093/bioinformatics/bts199 22543367 PMC3371832

[eva12804-bib-0043] Kengne, P. , Antonio‐Nkondjio, C. , Awono‐Ambene, H. P. , Simard, F. , Awolola, T. S. , & Fontenille, D. (2007). Molecular differentiation of three closely related members of the mosquito species complex, *Anopheles moucheti*, by mitochondrial and ribosomal DNA polymorphism. Medical and Veterinary Entomology, 21(2), 177–182. 17550437 10.1111/j.1365-2915.2007.00681.x

[eva12804-bib-0044] Kengne, P. , Awono‐Ambene, P. , Nkondjio, C. A. , Simard, F. , & Fontenille, D. (2003). Molecular identification of the *Anopheles nili* group of African malaria vectors. Medical and Veterinary Entomology, 17(1), 67–74. 10.1046/j.1365-2915.2003.00411.x 12680928

[eva12804-bib-0045] Letunic, I. , & Bork, P. (2007). Interactive Tree Of Life (iTOL): An online tool for phylogenetic tree display and annotation. Bioinformatics, 23(1), 127–128. 10.1093/bioinformatics/btl529 17050570

[eva12804-bib-0046] Li, S. J. , Ahmed, M. Z. , Lv, N. , Shi, P. Q. , Wang, X. M. , Huang, J. L. , & Qiu, B. L. (2017). Plant‐mediated horizontal transmission of Wolbachia between whiteflies. ISME Journal, 11(4), 1019–1028. 10.1038/ismej.2016.164 27935594 PMC5364347

[eva12804-bib-0047] Makanga, B. , Yangari, P. , Rahola, N. , Rougeron, V. , Elguero, E. , Boundenga, L. , … Paupy, C. (2016). Ape malaria transmission and potential for ape‐to‐human transfers in Africa. PNAS, 113(19), 5329–5334. 10.1073/pnas.1603008113 27071123 PMC4868493

[eva12804-bib-0048] McGraw, E. A. , & O'Neill, S. L. (2013). Beyond insecticides: New thinking on an ancient problem. Nature Reviews Microbiology, 11(3), 181–193. 23411863 10.1038/nrmicro2968

[eva12804-bib-0049] Moreira, L. A. , Iturbe‐Ormaetxe, I. , Jeffery, J. A. , Lu, G. , Pyke, A. T. , Hedges, L. M. , … O'Neill, S. L. (2009). A Wolbachia symbiont in *Aedes aegypti* limits infection with dengue, chikungunya, and plasmodium. Cell, 139(7), 1268–1278. 10.1016/j.cell.2009.11.042 20064373

[eva12804-bib-0050] Ndo, C. , Antonio‐Nkondjio, C. , Cohuet, A. , Ayala, D. , Kengne, P. , Morlais, I. , … Simard, F. (2010). Population genetic structure of the malaria vector *Anopheles nili* in sub‐Saharan Africa. Malaria Journal, 9, 161 10.1186/1475-2875-9-161 20540796 PMC2898787

[eva12804-bib-0051] Neafsey, D. E. , Waterhouse, R. M. , Abai, M. R. , Aganezov, S. S. , Alekseyev, M. A. , Allen, J. E. , … Besansky, N. J. (2015). Highly evolvable malaria vectors: The genomes of 16 Anopheles mosquitoes. Science, 347(6217), 43 10.1126/science.1258522 PMC4380271 25554792

[eva12804-bib-0052] Newby, G. , Bennett, A. , Larson, E. , Cotter, C. , Shretta, R. , Phillips, A. A. , & Feachem, R. G. A. (2016). The path to eradication: A progress report on the malaria‐eliminating countries. Lancet, 387(10029), 1775–1784. 27116283 10.1016/S0140-6736(16)00230-0

[eva12804-bib-0053] Niang, E. H. A. , Bassene, H. , Makoundou, P. , Fenollar, F. , Weill, M. , & Mediannikov, O. (2018). First report of natural Wolbachia infection in wild *Anopheles funestus* population in Senegal. Malaria Journal, 17(1), 408 10.1186/s12936-018-2559-z 30400987 PMC6219158

[eva12804-bib-0054] Olson, D. M. , Dinerstein, E. , Wikramanayake, E. D. , Burgess, N. D. , Powell, G. V. N. , Underwood, E. C. , … Kassem, K. R. (2001). Terrestrial ecoregions of the worlds: A new map of life on Earth. BioScience, 51(11), 933–938. 10.1641/0006-3568(2001)051[0933:teotwa]2.0.co;2

[eva12804-bib-0055] Osei‐Poku, J. , Han, C. , Mbogo, C. M. , & Jiggins, F. M. (2012). Identification of wolbachia strains in mosquito disease vectors. PLoS ONE, 7(11), e49922 10.1371/journal.pone.0049922 23185484 PMC3503815

[eva12804-bib-0056] Pates, H. , & Curtis, C. (2005). Mosquito behavior and vector control. Annual Review of Entomology, 50, 53–70. 10.1146/annurev.ento.50.071803.130439 15355233

[eva12804-bib-0057] Paupy, C. , Makanga, B. , Ollomo, B. , Rahola, N. , Durand, P. , Magnus, J. , … Prugnolle, F. (2013). Anopheles moucheti and *Anopheles vinckei* are candidate vectors of ape plasmodium parasites, including Plasmodium praefalciparum in Gabon. PLoS ONE, 8(2), e57294 10.1371/journal.pone.0057294 23437363 PMC3577705

[eva12804-bib-0058] Pombi, M. , Kengne, P. , Gimonneau, G. , Tene‐Fossog, B. , Ayala, D. , Kamdem, C. , … Costantini, C. (2017). Dissecting functional components of reproductive isolation among closely related sympatric species of the *Anopheles gambiae* complex. Evolutionary Applications, 10(10), 1102–1120. 10.1111/eva.12517 29151864 PMC5680640

[eva12804-bib-0059] Rahola, N. , Makanga, B. , Yangari, P. , Jiolle, D. , Fontenille, D. , Renaud, F. , … Paupy, C. (2014). Description of Anopheles gabonensis, a new species potentially involved in rodent malaria transmission in Gabon, Central Africa. Infection Genetics and Evolution, 28, 628–634. 10.1016/j.meegid.2014.05.012 24840150

[eva12804-bib-0060] Ranson, H. , & Lissenden, N. (2016). Insecticide resistance in African anopheles mosquitoes: A worsening situation that needs urgent action to maintain malaria control. Trends in Parasitology, 32(3), 187–196. 10.1016/j.pt.2015.11.010 26826784

[eva12804-bib-0061] Richardson, M. F. , Weinert, L. A. , Welch, J. J. , Linheiro, R. S. , Magwire, M. M. , Jiggins, F. M. , & Bergman, C. M. (2012). Population Genomics of the Wolbachia Endosymbiont in Drosophila melanogaster. PLOS Genetics, 8(12), e1003129 10.1371/journal.pgen.1003129 23284297 PMC3527207

[eva12804-bib-0062] Robert, V. , Ayala, D. , & Simard, F. (2017). Les anopheles In Duvallet G., Fontenille D. & Robert V. (Eds.), Entomologie médicale et vétérinaire (p. 687). Paris, France: IRD Editions.

[eva12804-bib-0063] Rossi, P. , Ricci, I. , Cappelli, A. , Damiani, C. , Ulissi, U. , Mancini, M. V. , … Favia, G. (2015). Mutual exclusion of Asaia and Wolbachia in the reproductive organs of mosquito vectors. Parasit Vectors, 8, 1–10. 10.1186/s13071-015-0888-0 25981386 PMC4445530

[eva12804-bib-0064] Santolamazza, F. , Mancini, E. , Simard, F. , Qi, Y. , Tu, Z. , & della Torre, A. (2008). Insertion polymorphisms of SINE200 retrotransposons within speciation islands of *Anopheles gambiae* molecular forms. Malaria Journal, 7, 163 10.1186/1475-2875-7-163 18724871 PMC2546427

[eva12804-bib-0065] Schmidt, T. L. , Barton, N. H. , Rašić, G. , Turley, A. P. , Montgomery, B. L. , Iturbe‐Ormaetxe, I. , … Turelli, M. (2017). Local introduction and heterogeneous spatial spread of dengue‐suppressing Wolbachia through an urban population of Aedes aegypti. PLOS Biology, 15(5), e2001894 10.1371/journal.pbio.2001894 28557993 PMC5448718

[eva12804-bib-0066] Shaw, W. R. , Marcenac, P. , Childs, L. M. , Buckee, C. O. , Baldini, F. , Sawadogo, S. P. , … Catteruccia, F. (2016). Wolbachia infections in natural Anopheles populations affect egg laying and negatively correlate with Plasmodium development. Nature Communications, 7, 11772 10.1038/ncomms11772 PMC4895022 27243367

[eva12804-bib-0067] Simon, F. , Siles‐Lucas, M. , Morchon, R. , Gonzalez‐Miguel, J. , Mellado, I. , Carreton, E. , & Montoya‐Alonso, J. A. (2012). Human and animal dirofilariasis: The emergence of a zoonotic. Mosaic. Clinical Microbiology Reviews, 25(3), 507–544. 10.1128/cmr.00012-12 22763636 PMC3416488

[eva12804-bib-0068] Sinkins, S. P. , Braig, H. R. , & Oneill, S. L. (1995). Wolbachia superinfections and the expression of cytoplasmic incompatibility. Proceedings of the Royal Society B‐Biological Sciences, 261(1362), 325–330. 10.1098/rspb.1995.0154 8587875

[eva12804-bib-0069] Stamatakis, A. (2006). *Phylogenetic models of rate heterogeneity: A high performance computing perspective* . Paper presented at the International Parallel and Distributed Processing Symposium, Rhodos, Greece

[eva12804-bib-0070] Stamatakis, A. (2014). RAxML version 8: A tool for phylogenetic analysis and post‐analysis of large phylogenies. Bioinformatics, 30(9), 1312–1313. 10.1093/bioinformatics/btu033 24451623 PMC3998144

[eva12804-bib-0071] Tsutsui, N. D. , Kauppinen, S. N. , Oyafuso, A. F. , & Grosberg, R. K. (2003). The distribution and evolutionary history of Wolbachia infection in native and introduced populations of the invasive argentine ant (Linepithema humile). Molecular Ecology, 12(11), 3057–3068. 10.1046/j.1365-294X.2003.01979.x 14629385

[eva12804-bib-0072] Walker, T. , Johnson, P. H. , Moreira, L. A. , Iturbe‐Ormaetxe, I. , Frentiu, F. D. , McMeniman, C. J. , … Hoffmann, A. A. (2011). The wMel Wolbachia strain blocks dengue and invades caged *Aedes aegypti* populations. Nature, 476(7361), 450–U101. 10.1038/nature10355 21866159

[eva12804-bib-0073] Werren, J. H. , Baldo, L. , & Clark, M. E. (2008). Wolbachia: Master manipulators of invertebrate biology. Nature Reviews Microbiology, 6(10), 741–751. 10.1038/nrmicro1969 18794912

[eva12804-bib-0074] Werren, J. H. , Zhang, W. , & Guo, L. R. (1995). Evolution and phylogeny of Wolbachia: Reproductive parasites of arthropods. Proceedings of the Royal Society B: Biological Sciences, 261(1360), 55–63. 10.1098/rspb.1995.0117 7644549

[eva12804-bib-0075] WHO (2015). Global technical strategy for malaria 2016–2030. Geneva, Switzerland: WHO.

[eva12804-bib-0076] WHO (2018). *World malaria report 2018* . Geneva, Switzerland: WHO

[eva12804-bib-0077] Wickham, H. (2009). ggplot2: Elegant graphics for data analysis. New York, NY: Springer Publishing Company, Incorporated.

[eva12804-bib-0078] Zele, F. , Nicot, A. , Berthomieu, A. , Weill, M. , Duron, O. , & Rivero, A. (2014). Wolbachia increases susceptibility to Plasmodium infection in a natural system. Proceedings of the Royal Society B‐Biological Sciences, 281(1779), 20132837 10.1098/rspb.2013.2837 PMC3924077 24500167

[eva12804-bib-0079] Zug, R. , & Hammerstein, P. (2012). Still a host of hosts for wolbachia: analysis of recent data suggests that 40% of terrestrial arthropod species are infected. PLoS ONE, 7(6), e38544 10.1371/journal.pone.0038544 22685581 PMC3369835

