# Supplementary material for: Natural Wolbachia infections are common in the major malaria vectors in Central Africa
Source: Evol Appl. 2019 Jun 11;12(8):1583–94. doi: 10.1111/eva.12804 (PMC6708434; doi:10.1111/eva.12804)
Supplement: Supplementary file 1 [file EVA-12-1583-s001.docx]

**Supplementary Information**

Natural *Wolbachia* infections are common in the major malaria vectors in Central Africa.

**Authors**

Diego Ayala^1,2,*^, Ousman Akone-Ella^2^, Nil Rahola^1,2^, Pierre Kengne^1^, Marc F. Ngangue^2,3^, Fabrice Mezeme^2^, Boris K. Makanga^2^, Carlo Costantini^1^, Frédéric Simard^1^, Franck Prugnolle^1^, Benjamin Roche^1,4^, Olivier Duron^1^ & Christophe Paupy^1^.

**Text**

**Appendix S1.** Mosquito taxonomic and molecular identification.

Based on morphological traits(Gillies & Coetzee, 1987), we first identified 19 mosquito species. This number increased to 25 after the results of the molecular analysis (see below). Specimens with morphological differences relative to *An. coustani* were pooled as *An. gr. coustani*. The richness of *Anopheles* species in Central Africa was previously documented(Ayala et al., 2009; Makanga et al., 2016; Paupy et al., 2013; Service, 1976). In general, sylvatic habitats (i.e., La Lopé (n=11) and Bakoumba (n=13)) showed higher diversity than anthropic areas (i.e., Cocobeach (n=1) and Libreville (n=1)), with the only exception of Franceville (n=11) (Fig. 1). Among the collected mosquitoes, many belonged to groups or complexes of morphologically indistinguishable species(Robert, Ayala, & Simard, 2017). Molecular tools (PCR-based diagnosis) have been developed for the major human malaria vectors in Africa. They confirmed the identification of *An. gambiae* and *An. coluzzii* from the *gambiae* complex*; An. funestus* from the *funestus* group*; An. nili* and *An carnevalei* from the *nili* complex*; and An. moucheti moucheti* (hereafter, *An. m. moucheti*) and *An. moucheti nigeriensis (*hereafter, *An. m. nigeriensis)* from the *moucheti* complex. However, for several mosquito specimens that were morphologically identified as *nili* and *moucheti,* this classification was not confirmed by PCR-based species diagnosis(Kengne et al., 2007; Kengne, Awono-Ambene, Nkondjio, Simard, & Fontenille, 2003). *COII* sequence analysis revealed that they might represent new non-described taxa. Therefore, these *An. nili* specimens were arbitrarily called *An. “GAB-1”*, and the non-classified *An.* *moucheti* samples were arbitrarily called *An. “GAB-2” and An. “GAB-3”* (*GAB-3* was exclusively found at Plateaux Batekes National Parc) (Supplementary Table 1). Additional morphological, ecological and molecular studies are required for the correct assignation of species status.

**Tables**

**Table S1.** Mosquitoes screened in this study and their accession numbers.

ID: Specimen identification name; Sites: Collection locations; Species: Morphological and molecular identification; COII_hap: COII haplotype for each species; COII: accession number for the cytochrome oxidase subunit II gene; 16S: accession number for the 16S rRNA gene; ftsZ: accession number for the filamenting temperature-sensitive mutant Z protein; fbpA: accession number the fructose-bisphosphate putative aldolase protein; coxA: accession number for the cytochrome c oxidase subunit I.

**Table S2.** Summary of the mosquitoes collected and screened in this study

**Table S3**. *Anopheles moucheti* F1 used to estimate vertical transmission.

**Table S4**. Genetic distances between *Wolbachia* strains (*16S*) and infected *Anopheles* species *(COII)*.

Genetic distances were estimated as the number of different bases between the sequences of each pair of infected *Anopheles* specimens (see Fig. S2).

**Figures**

**Figure S1**. Rooted maximum likelihood phylogeny of the filarial *Wolbachia* sequence isolated from one *An. coustani* specimen.

The tree was inferred with RAxML (Stamatakis, 2014) using the sequence of the filarial *COII* fragment amplified from the *An. coustani* specimen BNG78 (in blue) and public sequences (NCBI) and rooted with *Brugia malayi* as outgroup. The black dot on the branch indicates a bootstrap value >70% from 1000 replicates.

**Figure S2**. Scatterplot showing the genetic distances between *Wolbachia* strains (*16S*) and infected *Anopheles* species *(COII)*.

Genetic distances were estimated as the number of different bases between the sequences of each pair of infected *Anopheles* specimens. The smoothed conditional mean (read line) and the 95% confidence intervals (blue area) were plotted using the smoothing “gam” function of the ggplot2 library (Wickham, 2009).

**Figure S3**. Probability of detecting *Wolbachia* infection.

The probability was estimated for each sample size and infection prevalence value. The probability of correct estimation follows a grey gradient. The dashed line indicates the 90% probability of infection detection. We estimated that a sample size of 60 individuals per species was needed to quantify correctly a prevalence lower than 15%, with a probability of 95%. As our sample size mostly varied from 1 to 58 individuals for each species, with few exceptions in which sample size was lower than five individuals, the probability of a correct estimation of *Wolbachia* prevalence ranged between 68% and 93%.

**References**

Ayala, D., Costantini, C., Ose, K., Kamdem, G., Antonio-Nkondjio, C., Agbor, J.-P., . . . Simard, F. (2009). Habitat suitability and ecological niche profile of major malaria vectors in Cameroon. *Malar J, 8*(1), 307.

Gillies, M. T., & Coetzee, M. C. (1987). *A Supplement to the Anophelinae of Africa South of the Sahara (Afrotropical region)*. Johannesburg: The South African Institute for Medical Research.

Kengne, P., Antonio-Nkondjio, C., Awono-Ambene, H. P., Simard, F., Awolola, T. S., & Fontenille, D. (2007). Molecular differentiation of three closely related members of the mosquito species complex, *Anopheles moucheti*, by mitochondrial and ribosomal DNA polymorphism. *Medical and veterinary entomology, 21*(2), 177-182.

Kengne, P., Awono-Ambene, P., Nkondjio, C. A., Simard, F., & Fontenille, D. (2003). Molecular identification of the *Anopheles nili* group of African malaria vectors. *Medical and veterinary entomology, 17*(1), 67-74. doi:doi:10.1046/j.1365-2915.2003.00411.x

Makanga, B., Yangari, P., Rahola, N., Rougeron, V., Elguero, E., Boundenga, L., . . . Paupy, C. (2016). Ape malaria transmission and potential for ape-to-human transfers in Africa. *PNAS, 113*(19), 5329-5334. doi:10.1073/pnas.1603008113

Paupy, C., Makanga, B., Ollomo, B., Rahola, N., Durand, P., Magnus, J., . . . Prugnolle, F. (2013). Anopheles moucheti and Anopheles vinckei Are Candidate Vectors of Ape Plasmodium Parasites, Including Plasmodium praefalciparum in Gabon. *PLoS ONE, 8*(2). doi:e57294

10.1371/journal.pone.0057294

Robert, V., Ayala, D., & Simard, F. (2017). Les Anopheles. In G. Duvallet, D. Fontenille, & V. Robert (Eds.), *Entomologie médicale et vétérinaire*. Paris: IRD Editions.

Service, M. W. (1976). Contribution to the knowledge of the mosquitoes (Diptera, Culicidae) of Gabon. *Cahiers ORSTOM, Series Entomologique Medicale et Parasitologie, XIV*(3), 259-263.

Stamatakis, A. (2014). RAxML version 8: a tool for phylogenetic analysis and post-analysis of large phylogenies. *Bioinformatics, 30*(9), 1312-1313. doi:10.1093/bioinformatics/btu033

Wickham, H. (2009). *ggplot2: Elegant Graphics for Data Analysis*: Springer Publishing Company, Incorporated.
